# Management of ventricular tachycardias: insights on centre settings, procedural workflow, endpoints, and implementation of guidelines—results from an EHRA survey

**DOI:** 10.1093/europace/euae030

**Published:** 2024-02-14

**Authors:** Arian Sultan, Piotr Futyma, Andreas Metzner, Ante Anic, Sergio Richter, Laurent Roten, Patrick Badertscher, Giulio Conte, Julian K R Chun

**Affiliations:** Department of Electrophysiology, Heart Centre University Hospital Cologne, Germany; St. Joseph’s Heart Rhythm Centre, Rzeszów, Poland; Medical College, University of Rzeszów, Rzeszów, Poland; Department of Cardiology, University Heart and Vascular Centre Hamburg, University Medical Centre Hamburg-Eppendorf, Hamburg, Germany; Department for Cardiovascular Diseases, University Hospital Centre Split, Spilt, Croatia; Division of Electrophysiology, Department of Internal Medicine and Cardiology, Heart Centre Dresden, University Hospital, Technische Universität Dresden, Dresden, Germany; Inselspital-Bern University Hospital, Department of Electrophysiology University of Bern, Bern, Switzerland; Inselspital-Bern University Hospital, Department of Electrophysiology University of Bern, Bern, Switzerland; Division of Cardiology, Cardiocentro Ticino (CCT), Lugano, Switzerland; Cardioangiologisches Centrum Bethanien, Agaplesion Markus Krankenhaus, Frankfurt am Main, Germany

**Keywords:** VT ablation, Sudden cardiac death, Guidelines, Medical therapy, Advanced ablation

## Abstract

Ventricular tachycardia (VT), and its occurrence, is still one of the main reasons for sudden cardiac death and, therefore, for increased mortality and morbidity foremost in patients with structural heart [Kahle A-K, Jungen C, Alken F-A, Scherschel K, Willems S, Pürerfellner H *et al.* Management of ventricular tachycardia in patients with ischaemic cardiomyopathy: contemporary armamentarium. *Europace* 2022;**24**:538–51]. Catheter ablation has become a safe and effective treatment option in patients with recurrent VT [Cronin EM, Bogun FM, Maury P, Peichl P, Chen M, Namboodiri N *et al.* 2019 HRS/EHRA/APHRS/LAHRS expert consensus statement on catheter ablation of ventricular arrhythmias. *Heart Rhythm* 2020;**17**:e2–154]. Previous and current guidelines provide guidance on indication for VT ablation and risk assessment and evaluation of underlying disease. However, no uniform recommendation is provided regarding procedural strategies, timing of ablation, and centre setting. Therefore, these specifics seem to differ largely, and recent data are sparse. This physician-based European Heart Rhythm Association survey aims to deliver insights on not only infrastructural settings but also procedural specifics, applied technologies, ablation strategies, and procedural endpoints. Therefore, these findings might deliver a real-world scenario of VT management and potentially are of guidance for other centres.

## Graphical Abstract

**Graphical Abstract:**
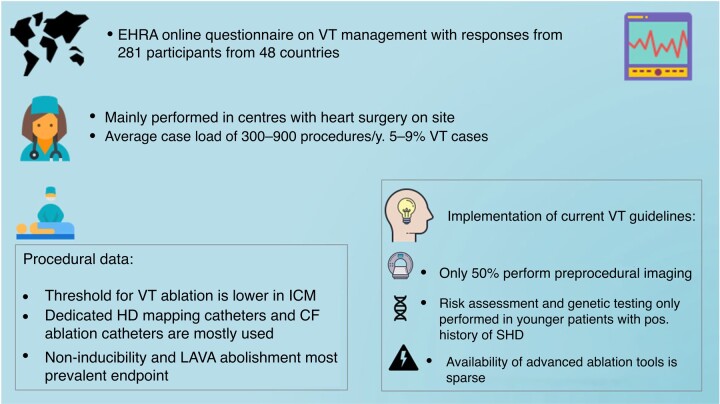

## Introduction

Approximately 10–20% of all deaths are caused by a sudden cardiac death (SCD) due to ventricular arrhythmias. Implantation of implantable cardioverter defibrillators (ICDs) and catheter ablation (CA) not only might prevent SCD but also reduce the number of ICD interventions.^1,2^ Therefore, CA has become indispensable in the setting of ventricular arrhythmia.^3^ Despite tremendous development in the field of CA for ventricular arrhythmia, improved evaluation and understanding of mechanisms, and constantly updated guidelines,^1,4^ data on implementation of the latter in daily workflow are sparse. Also, information on centre specificities such as personnel, access to novel technologies, and ventricular tachycardia (VT) management and ablation strategy is lacking.^5,6^ Therefore, this survey was carried out by the European Heart Rhythm Association (EHRA) initiated by the Scientific Initiative Committee (SIC) to investigate the real-world management of VT and the current adaptation and implementation of the new guidelines and ablation modalities. Hence, this survey will provide more detailed information on centre specificities across Europe and associated non-European countries. Aside from gathering information on before-mentioned topics, this survey also aimed how frequent and in which setting additional technologies (e.g. pre-procedural imaging, assist devices, and alternative ablation modalities) and new mapping options are used. We also wanted to provide insights on ablation strategies and pre- and post-ablation management, which are not completely covered by the current guidelines and differ largely between centres and countries. Furthermore, the newly introduced importance on risk stratification especially in the setting of structural heart disease in non-ischaemic cardiomyopathy (NICM), such as dilatative cardiomyopathy (DCM) and hypokinetic non-dilative cardiomyopathy (HNDCM), has been evaluated in this survey. In summary, this survey provides an overview of real-life VT management, ablation strategies and procedural settings down to catheter types, mapping technologies, energy settings, ablation endpoints, antiarrhythmic drug (AAD) regime, and risk assessment for SCD.

This survey will not only give an overview of daily VT practice and reflect the status of implementation of current VT guidelines but hopefully will lead to knowledge exchange and enabling less equipped centres to network for optimal patient treatment across European Union, Europe, Middle East and Africa (EMEA), Asia and Latinamerica from a practical perspective.

## Methods

This survey was an online questionnaire created by the EHRA SIC sent out by EHRA. It was then distributed via social media and national cardiac and electrophysiologic societies and their members. The survey was accessible for 6 weeks (starting April 2023), and its participation was physician based, anonymous, and voluntary (Supplementary material online).

The survey was divided into two sections and consisted of 42 questions. The first part was aiming at more general aspects of VT management such as number of VT ablations, facilities, acute treatment of VT, timing of ablation, access to novel technologies, and imaging. Also, implementation of new guideline recommendations for e.g. risk assessment^1^ was queried. The second part of the survey focused on procedural aspects: ablation strategies and endpoints, ablation modalities, use of advanced ablation techniques, and the use of assist devices for VT ablation.

## Results

### General aspects

**Figure 1:**
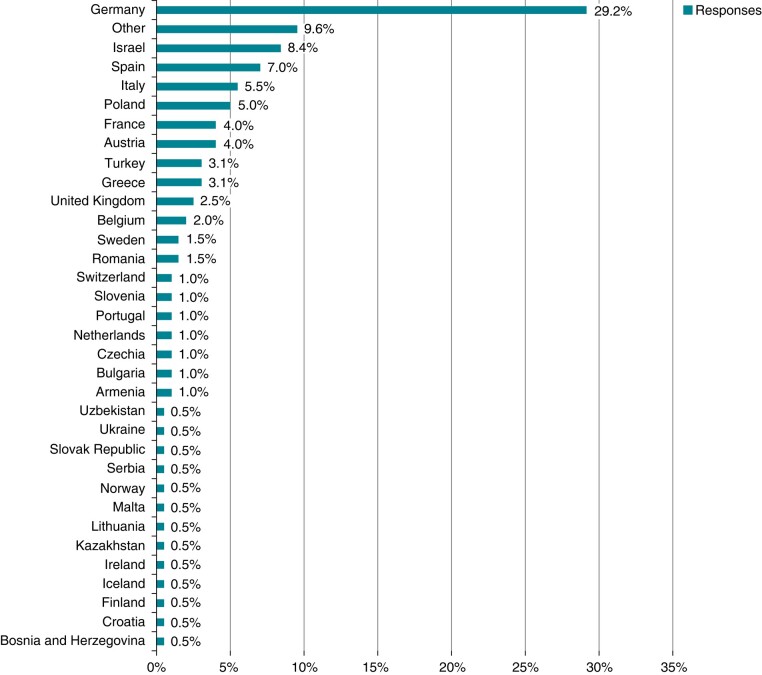
Response by countries.

After a period of 6 weeks, a total of 281 participants from 48 different countries replied to the survey. Although the survey was distributed through different online outlets, most responses were obtained in Europe, in particular from Germany (29%), Spain (7%), and Italy (6%). Outside the European Union but within ‘EMEA countries, Israel (7%) and Turkey (3%) were the most responsive (*Figure 1*). The term ‘others’ listed in *Figure 1* include countries in Latin and Central America (e.g. Argentina and Panama), East Asia (e.g. Singapore and Indonesia), and the Middle East (e.g. Yemen). The majority of participants (45%) indicated to perform 300–900 CA procedures in general at their centres per year (y). Almost a quarter (22%) indicated to perform 100–300 CA/y. Only 17% perform >1000 CA/y. Of note, up to 8% indicated to perform <50 CA/y in total. On average, 52 VT procedures including outflow tract VT ablation are performed per year of which 44% are in the setting of ischaemic cardiomyopathy (ICM). The survey revealed that, on average, 3 physicians per centre perform VT ablation on a regular basis.

### Centre settings

The predominant work setting reported was university hospitals in 67%. In 33%, participants work in a non-university hospital or private setting. In 77% of centres, heart surgery is available onsite.

In 81%, circulatory support systems such as extra-corporal membrane oxygenation (ECMO) and Impella are available at any time. One-third (30%) reported to have a dedicated VT storm unit or some sort of 24-h VT management service.

### Acute ventricular tachycardia management, pre-procedural imaging, and pre-procedural settings

For acute treatment of VT or in the case of a VT storm, participants were asked to report the preferred AAD regime in a ranked order and/or the use of additional interventions such as deep sedation or blockade of the stellate ganglion. Of note, not all listed AADs are equally available in all countries, or availability is limited to either oral or intravenous administration.

**Figure 2:**
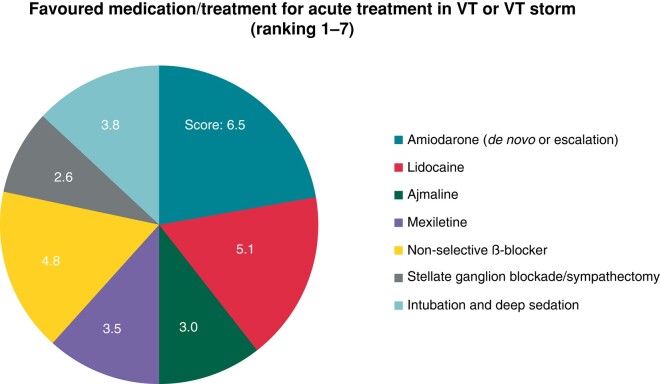
Distribution of used AADs and/or adjunctive treatments to treat VT or VT storm.

Amiodarone and lidocaine were the most frequent used AADs. However, non-selective β-blockers such as propranolol were also frequently used (*Figure 2*).

### Imaging and visualization

#### CMR and CT

The role of pre-procedural imaging has grown within the last years. More than half reported to obtain cardiomagnetic resonance (CMR)/LGE-CMR routinely before VT ablation (55.4%) and more than one-third (38.4%) perform computer tomography (CT)/3D CT including visualization of coronaries.

When asked to report if any specific software for image processing was used, four participants reported the use of ADAS3D software (Adas3D Medical SL, Barcelona, Spain) for CMR and five used inHEART software (inHEART Medical, Pessac, France) for CT. One centre reported on 3D rotational ventriculography reconstruction using the Philips EP Navigator system. The survey revealed that all 3D pre-procedural imaging is implemented into obtained electroanatomic maps (EAMs) during the procedure in 59.2%. To exclude coronary artery disease or progression of the latter, 61.5% of participants always perform coronary angiography before VT ablation.

#### Use of intracardiac echo

A total of 37% answered the question if intracardiac echo (ICE) was available or used at their centre. Of these, 70.9% employ ICE mostly in the setting of papillary muscle VT. Some centres use ICE in every procedure (see details in the table below, *Table 1*).

**Table 1:** Indication for the use of ICE in VT and PVC ablation

Papillary muscle	41%
Moderator band	6.4%
Outflow tract/aortic cusp	2.6%
To guide transseptal puncture	11.5%
In all cases	29.5%

#### Pre-procedural setting—oral anticoagulation and sedation

In patients scheduled for VT or premature ventricular contraction (PVC) ablation, 63.2% stop pre-existing oral anticoagulation (OAC). Most centres report to stop OAC 6–48 h before procedure.

The vast majority (74.6%) of procedures are performed in deep analgo-sedation. Approximately a quarter of reporting physicians (23.4%) use general anaesthesia in all VT procedures.

#### Timing of ventricular tachycardia ablation and advanced ablation options

**Figure 3:**
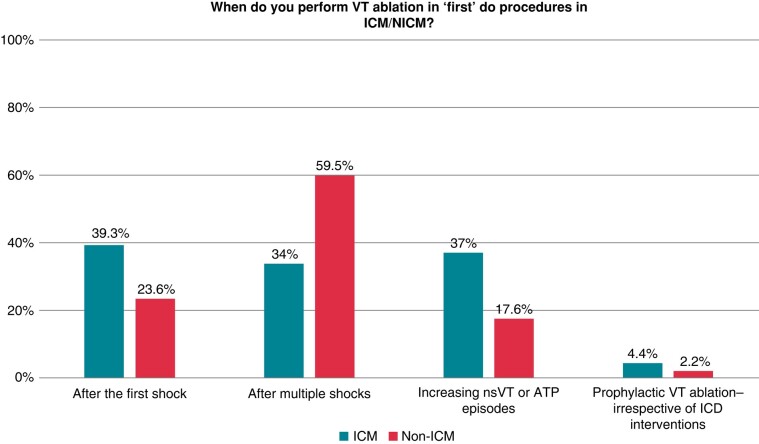
Timing of first ablation attempt.

Participants were asked to report on timing for VT ablation in the setting of ICM and NICM. Only a small proportion answered to perform ‘prophylactic VT ablation’ before occurrence of first ICD intervention. The vast majority only performed ablation after previous ICD interventions. However, the survey revealed a difference between ICM and NICM (*Figure 3*). In NICM patients, VT ablation is mostly performed after several ICD shocks, whereas in ICM, the threshold for VT ablation is lower (24% vs. 39% after the first shock).

**Figure 4:**
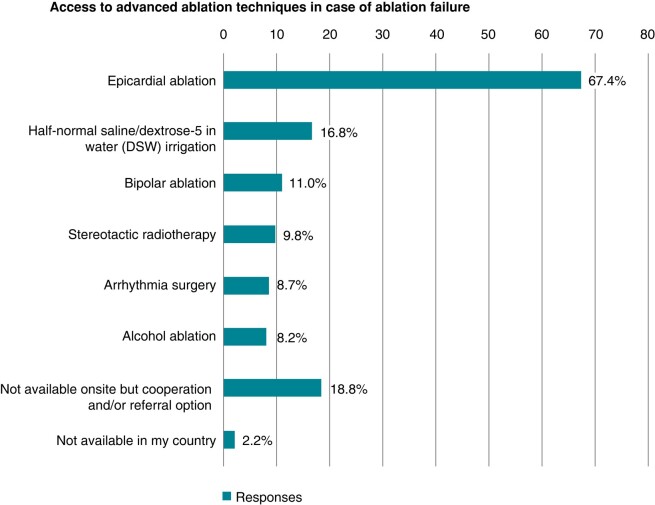
Use and/or access to advanced ablation techniques if conventional ablation failed.

Furthermore, information was obtained if centres would have access to alternative or advanced ablation techniques. Either performing the latter themselves or having transferal options. Multiple answers were possible. The most available advanced ablation option is the use of epicardial VT ablation or different irrigation solutions enhancing current flow into the tissue. Complete results are displayed below (*Figure 4*).

### Specifics of ventricular tachycardia ablation

#### Access

Participants (41.4%) answered to use ultrasound-guided groin puncture. A simultaneous retrograde aortic access as well as transeptal access to the left ventricle is obtained in most cases (41.9%). In 38.6%, a solely transseptal approach is obtained. In 19.6%, an aortic access only is used. An epicardial access beforehand was obtained in 18.6% based on electrocardiogram criteria suggesting an epicardial origin, in 13.8% in NICM patients only, and in 21% of the latter in a first VT ablation attempt. The vast majority, 53.3%, reported to use an epicardial access only after failed previous endocardial ablation.

#### Procedural workflow, ablation settings, and procedural endpoints

##### Workflow

Participants were asked to report on their procedural workflow regarding EAM, which mapping and ablation catheters are routinely used, and the location of ventricular stimulation for VT induction. Also, if an isochronal late activation mapping (ILAM),^7,8^ decremental evoked potential mapping (DEEP),^9,10^ or local abnormal ventricular activity (LAVA)^11^ mapping guided ablation approach was executed routinely. Results are summarized in *Figure 6*.

##### Catheter and energy settings

For more ablation specifics, regularly used ablation catheter and information on power settings were evaluated. The majority of participants (78.8%) report to use irrigated contact force catheters regularly. Some (15.6%) use non-contact force irrigated catheter for VT ablation. An energy setting ranging between 30 and 50 W was most prevalent (78.8%). In 12.8%, energy was uptitrated to 50 W. In 3.9%, 60 W was used. New energy sources like pulsed field ablation (PFA) and ultra-low cryo were used in 1.7 and 0.6%, respectively. The indicated power setting for epicardial ablation ranged from 30 to 60 W.

Furthermore, the questionnaire also evaluated the use of ablation indices such as lesion size index (LSI) and ablation index (AI),^12,13^ although being more commonly used and validated for the atrium. More than half of participants, 58.6%, answered to use either LSI or AI in VT ablation. Reported values for LSI ranged from 3 to 8 and for AI from 500 to 1000.

##### Procedural endpoints

Physicians were asked to report on their lesion assessment and procedural endpoints for VT ablation. To reflect actual real-world VT proceedings, multiple answers were possible.

**Figure 5:**
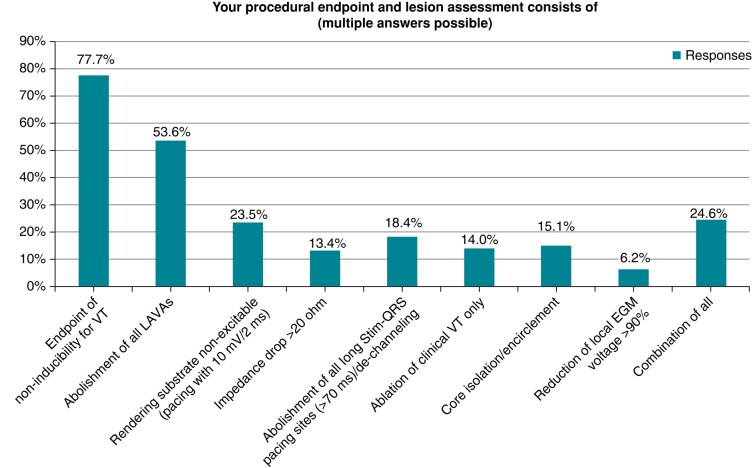
Distribution of procedural endpoints in VT ablation.

Results on all reported procedural endpoints are summarized in *Figure 5*. Non-inducibility of VT and abolishment of all LAVAs seem to be the most frequent endpoints in VT ablation.

In addition to the previous question on access to advanced ablation techniques (*Figure 4*), we also asked the participants when they would apply these options, if applicable. The majority (50.9%) uses these technologies only after previously failed ablation (second procedure). More than one-third (30.8%) reports not to have access to advanced ablation techniques at their centres. If VT documentation or underlying disease would suggest an epicardial origin, 11.7% would apply the forementioned advanced ablation techniques during the first ablation attempt.

##### Use of assist devices

Physicians were asked on which basis the decision to employ an assist device is made. The main factor triggering the use of an assist device was an impaired ejection fraction in most centres (56.3%). One-quarter (25.8%) reported to use assist devices in patients in VT storm. Decision-making based on scores like the I-VT score/PAINESD score was reported in 22.2%.

### Post-ablation management—vessel closure, oral anticoagulation, and antiarrhythmic drugs

#### Venous and arterial closure

For groin access closure in 40%, a Z-suture is used. For closure of arterial access in 40% of centres, specific closure devices are used (e.g. AngioSeal). Closure devices for venous access are used in 2.2% of centres. In 18.8% of centres, access closure is obtained by manual compression only.

#### Post-procedural oral anticoagulation management

Assessment of post-procedural OAC management (if no other indication for OAC therapy was present, e.g. atrial fibrillation) revealed that, in 35.0%, ASS monotherapy and, in 45.2%, a DOAC therapy (mostly apixaban) are prescribed after VT ablation.

In 4.5%, clopidogrel monotherapy is used. Duration of OAC therapy varied from 4 to 52 weeks. In the remaining 15.3%, no OAC is used after VT ablation. Only a few reported on using low-weight-molecular heparin during the hospital stay.

#### Management of antiarrhythmic drugs

Physicians were asked if they always discontinue any pre-existing AAD if VT ablation is acutely considered successful. One-third (33.2%) answered to always stop AAD after a successful VT ablation, and 13% answered to sometimes stop AAD. Despite a successful VT ablation, 65.4% report to always continue AADs for at least 3 months and 9.7% to sometimes continue AADs.

#### Wearable cardioverter defibrillator in preserved ejection fraction, risk assessment, and genetic testing

The questionnaire also evaluated if physicians tend to equip their patients with a wearable cardioverter defibrillator (WCD, LifeVest) after VT ablation in the setting of preserved ejection fraction. This was the case in 16.8% of participants.

Acknowledging the new guideline recommendations, the role of risk assessment and genetic testing was elicited in this survey. Asking if EP studies are performed for risk assessment of VT or SCD, the vast majority, 64.3%, perform electrophysiological study (EPS) only in symptomatic patients (dizziness or syncope) with known structural heart disease. In 17%, EPS is only performed after the first episode of sustained monomorphic VT. In 19.2%, physicians stated not to perform EPS for risk assessment. Regarding genetic testing for risk stratification or evaluation of underlying disease, most participants, 64.3%, only perform genetic testing in patients <50 years of age suffering from DCM or HNDCM with a positive family history for the latter or SCD; 22.4% report to perform genetic testing in all patients suffering from DCM or HNDCM. Fewer participants (15.6%) test in DCM/HNDCM patients with AV conduction abnormalities; 11.2% state to find genetic testing not valuable in this particular setting.

## Discussion

Catheter ablation has become a cornerstone in the treatment of recurrent VT and has been proven to be superior to AAD therapy.^3,14,15^ Although guidelines of VT management are constantly updated,^1,4^ data on implementation of the latter and information on real-world management of VT and VT ablation specifics are sparse. Furthermore, details on ablation specificities, such as mapping, and ablation strategies or lesion assessment are not completely covered by the current guidelines. This survey aimed to reflect real-world VT management and to provide daily practice data, potentially facilitating VT ablation in different settings possibly improving outcome, safety, and patient care.

Main findings of this survey are the following:

1. Ventricular tachycardia ablation is mainly performed in university hospitals. Most participants report to have heart surgery on site. A case load of 300–900 procedures of which 50 VT ablations are performed per year.
2. More than 50% obtain CMR/LGE-CMR routinely before VT ablation.
3. No prophylactic VT ablation is performed. However, in ICM, the threshold for VT ablation is lower than in NICM.
4. Availability or use of advanced ablation techniques is sparse. Epicardial ablation and half-normal saline irrigation are used most frequently as additional ablation techniques.
5. For mapping, 85% use a high density (HD) mapping catheter. For VT ablation, contact force-enabled catheters are employed in most cases.
6. Non-inducibility of VT and abolishment of all LAVA are the predominantly reported endpoints in VT ablation.
7. Risk assessment by EPS or genetic testing is only performed in younger symptomatic patients with positive history of structural heart disease.

### Centre settings, acute ventricular tachycardia management, and access to advanced ablation technologies

#### Centres

This survey conducted by the SIC and distributed by EHRA revealed that the majority of participants performing VT ablation have a significant overall case load with heart surgery on site. In comparison to the previous survey by Tilz *et al.*,^16^ the number of VT ablation increased over time from <50 VT ablations/centre per year to now >50 VT ablations/centre without increasing the total number of centre volume significantly.

Of note, our survey indicates that VT ablation is still performed in centres with an overall case load of <50/y. This finding is worrying considering reported correlation of outcome and centre volume for less complex procedures and patients as in the setting of atrial fibrillation and pulmonary vein isolation.^17^ However, in this survey for the first time, one-third of physicians report to have a dedicated 24-h VT unit or some kind of 24-h service. These findings are slightly different to the recent survey on VT storm in Europe reporting a 24-h availability in only 16.5% of the centres.^18^

#### Ventricular tachycardia management

The acute pharmacological VT management confirmed previous findings of amiodarone and lidocaine being administered most frequently. However, assumingly to recent publication showing superior efficacy in the treatment of VT storm, non-selective β-blockers are used increasingly as indicated by the participants.^1,19^

Of note, recently, a survey has been published focusing more thoroughly on the management of electrical storm.^18^

Although only a few stated to perform prophylactic CA, which is not supported by current data,^20^ general timing of VT ablation as answered in this survey has slightly changed over time. In patients with ICM, the threshold for VT ablation is lower as compared to NICM. In the setting of ICM, 39.3% of physicians offer VT ablation already after the first shock and 37% already in the case of increasing antitachycardia pacing (ATP) therapies. In NICM, physicians seem to await recurrent ICD shocks. Only 23.6% would perform VT ablation after the first shock. These findings nicely reflect the implementation of the current guidelines from the ESC and AHA/ACC/HRS for VT treatment in ICM and NICM.^1,21^ More detailed data regarding time interval between ischaemic event and necessity of first ablation would be desirable.

Also emphasized by the current VT guidelines, the survey revealed that 55.4% of participants routinely obtain CMR or CT imaging before VT ablation. This is an increase of >20% as compared to the survey by Tilz *et al*.^16^ Interestingly, only two-thirds integrate obtained CMR or CT imaging into their intraprocedural obtained EAM. A possible explanation might be the significant costs of dedicated imaging software to display and integrate VT substrate.

#### Ablation specifics

Within the last years, new ablation modalities such as bipolar ablation, the use of half-normal saline irrigation, and stereotactic ablation have been introduced to improve VT ablation and long-term outcome.^22–27^

This survey, for the first time, evaluated availability and application of latter. Interestingly, the most frequently employed advanced ablation technique is an epicardial ablation approach or the use of half-normal saline irrigation.

Only 11.7% indicated to apply these options in a first do procedure despite the fact that certain VT entities and their 3D substrate potentially profit from a more advanced ablation approach in the first procedure (e.g. septal^28,29^ VT and VT in Brugada).^23,30^ Of note, up to 20% reported not to have access to advanced ablation techniques at all. Therefore, this survey reveals the need for a VT network in less equipped centres or regions to facilitate potential cooperation and improve optimal VT ablation outcome.

**Figure 6:**
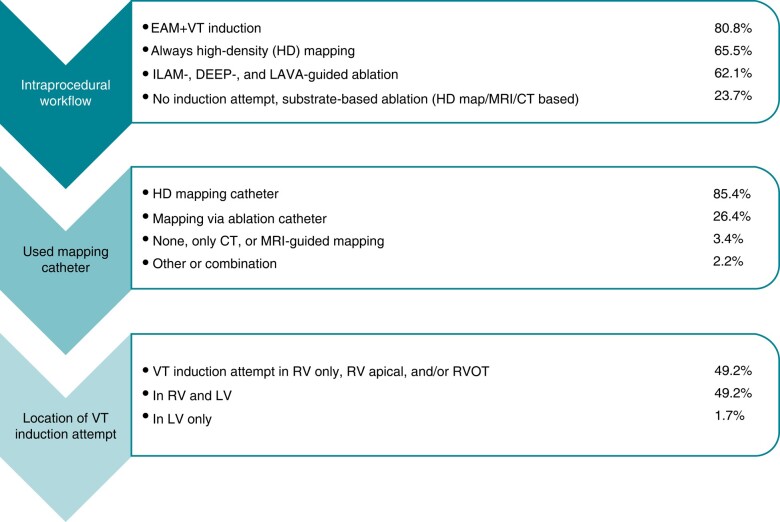
Workflow for VT ablation: Procedural strategy, mapping and used tools.

The largest variety was observed for the intraprocedural workflow (*Figure 6*). Although conventional EAM and VT induction are still the main approaches, new ablation strategies such as imaging-guided or functional substrate-guided (ILAM and DEEP) ablation are on the march.^31,32^ Ongoing studies (InEurHeart, NCT05225935) might deliver further evidence to unify guideline recommendations on ablation modality, therefore leading to shorter and safer VT procedures. However, despite all development in the field of VT ablation, non-inducibility of VT and substrate homogenization still seem to be the widely accepted endpoints.

The use of assist devices in haemodynamically impaired patients or in fast VT has been discussed in previous studies. Risk scores such as the PAINESD score or the I-VT score are recommended to discern patients to undergo VT ablation in the setting of an assist device.^33,34^ However, this survey revealed that most participants rather rely on left ventricular ejection fraction (LVEF) when deciding to use an assist device. Recent studies support that forementioned scores do not perform well in distinct VT population, and therefore, severely impaired LVEF might serve more accurately as a procedural risk predictor.^35^

#### Post-procedural management

Compared to the survey from 2018,^16^ data from DOAC after VT studies are apparently more frequently implemented.^36^ Therefore, 45.2% of physicians indicated to prescribe DOACs after VT ablation for a minimum of 4 weeks.

Although the use of wearable defibrillator (LifeVest) should be restricted to distinct populations, this survey revealed that 16.8% of physicians equip their patients with the latter despite successful VT ablation in the setting of preserved ejection fraction. This interesting finding is not supported by the current guidelines or previous reviews.^1,37^

#### Risk assessment and genetic testing

As a novelty, the current guideline emphasizes on the importance of risk assessment and genetic testing foremost in patients with symptoms in the setting of structural heart disease and/or patients suffering from DCM/HNDCM. According to the percentages indicated in the survey for EPS and genetic testing, this is potentially one of the topics that needs more awareness and therefore be improved timely, since <20% genetic testing is far too low especially in patients with DCM/HNDCM incorporating a huge variety of underlying disease (e.g. laminopathy) with severe risk of SCD.^38–40^

### Limitations

The nature of the surveys and its recipients might lead to a selection bias since due to the outlet and distribution of the latter, only scientifically interested physicians participate. This potentially reflects a narrower real-world scenario. Also, it should be mentioned that many responses were received from one country (Germany). Therefore, the before-mentioned biases might be pronounced. In the future, repeat surveys specifically comparing countries could be of potential interest and reflect more on healthcare system differences. However, in this survey, the large number of participating physicians and countries, also outside Europe, is of value.

## Conclusions

The current survey demonstrates that the management of VT has already implemented quite well some of the new aspects of the new VT guidelines. However, this survey also revealed topics that need to be improved in a timely manner. This survey is intended to enable improvement of VT management by sharing the reported insights on VT management and facilitate collaboration for improved patient outcome.

## Supplementary Material

euae030_Supplementary_Data

## Data Availability

All relevant data are within the manuscript and its supporting information files.

## References

[1] Zeppenfeld K , Tfelt-Hansen J, de Riva M, Winkel BG, Behr ER, Blom NA et al 2022 ESC Guidelines for the management of patients with ventricular arrhythmias and the prevention of sudden cardiac death. Eur Heart J 2022;43:3997–4126 . 36017572 10.1093/eurheartj/ehac262

[2] Lynge TH , Risgaard B, Banner J, Nielsen JL, Jespersen T, Stampe NK et al Nationwide burden of sudden cardiac death: a study of 54,028 deaths in Denmark. Heart Rhythm 2021;18:1657–65. 33965606 10.1016/j.hrthm.2021.05.005

[3] Natale A , Zeppenfeld K, Della BP, Liu X, Sabbag A, Santangeli P et al Twenty-five years of catheter ablation of ventricular tachycardia: a look back and a look forward. Europace 2023;25:euad225. 10.1093/europace/euad225 PMC10451002 37622589

[4] Cronin EM , Bogun FM, Maury P, Peichl P, Chen M, Namboodiri N et al 2019 HRS/EHRA/APHRS/LAHRS expert consensus statement on catheter ablation of ventricular arrhythmias. Europace 2019;21:1143–4. 31075787 10.1093/europace/euz132 PMC7967791

[5] Kahle A-K , Jungen C, Alken F-A, Scherschel K, Willems S, Pürerfellner H et al Management of ventricular tachycardia in patients with ischaemic cardiomyopathy: contemporary armamentarium. Europace 2022;24:538–51. 34967892 10.1093/europace/euab274

[6] Cronin EM , Bogun FM, Maury P, Peichl P, Chen M, Namboodiri N et al 2019 HRS/EHRA/APHRS/LAHRS expert consensus statement on catheter ablation of ventricular arrhythmias. Heart Rhythm 2020;17:e2–154. 31085023 10.1016/j.hrthm.2019.03.002 PMC8453449

[7] Aziz Z , Shatz D, Raiman M, Upadhyay GA, Beaser AD, Besser SA et al Targeted ablation of ventricular tachycardia guided by wavefront discontinuities during sinus rhythm. Circulation 2019;140:1383–97. 31533463 10.1161/CIRCULATIONAHA.119.042423

[8] Cabrera Borrego E , Sancez Millan P, Constan De La Revilla E, Tercedor Sanchez L, Alvarez Lopez M. Omnipolar technology in ventricular tachycardia ablation: activation and substrate mapping with late activation isochrone analysis under extrastimulus protocol. Europace 2023;25:euad122.311.

[9] Jackson N , Gizurarson S, Viswanathan K, King B, Massé S, Kusha M et al Decrement evoked potential mapping. Circ Arrhythm Electrophysiol 2015;8:1433–42. 26480929 10.1161/CIRCEP.115.003083

[10] Al-Sheikhli J , Winter J, Luque IR, Lambiase PD, Orini M, Porta-Sánchez A et al Optimization of decrementing evoked potential mapping for functional substrate identification in ischaemic ventricular tachycardia ablation. Europace 2023;25:euad092. 10.1093/europace/euad092 PMC10228600 37032650

[11] Jaïs P , Maury P, Khairy P, Sacher F, Nault I, Komatsu Y et al Elimination of local abnormal ventricular activities. Circulation 2012;125:2184–96. 22492578 10.1161/CIRCULATIONAHA.111.043216

[12] Bates AP , Paisey J, Yue A, Banks P, Roberts PR, Ullah W. Radiofrequency ablation of the diseased human left ventricle. JACC Clin Electrophysiol 2023;9:330–40. 36371330 10.1016/j.jacep.2022.10.001

[13] Casella M , Gasperetti A, Gianni C, Zucchelli G, Notarstefano P, Al-Ahmad A et al Ablation index as a predictor of long-term efficacy in premature ventricular complex ablation: a regional target value analysis. Heart Rhythm 2019;16:888–95. 30616020 10.1016/j.hrthm.2019.01.005

[14] Sapp JL , Wells GA, Parkash R, Stevenson WG, Blier L, Sarrazin J-F et al Ventricular tachycardia ablation versus escalation of antiarrhythmic drugs. N Engl J Med 2016;375:111–21. 27149033 10.1056/NEJMoa1513614

[15] Arenal Á , Ávila P, Jiménez-Candil J, Tercedor L, Calvo D, Arribas F et al Substrate ablation vs antiarrhythmic drug therapy for symptomatic ventricular tachycardia. J Am Coll Cardiol 2022;79:1441–53. 35422240 10.1016/j.jacc.2022.01.050

[16] Tilz RR , Lenarczyk R, Scherr D, Haugaa KH, Iliodromitis K, Pürerfellner H et al Management of ventricular tachycardia in the ablation era: results of the European Heart Rhythm Association Survey. Europace 2018;20:209–13. 29186419 10.1093/europace/eux332

[17] Deshmukh A , Patel NJ, Pant S, Shah N, Chothani A, Mehta K et al In-hospital complications associated with catheter ablation of atrial fibrillation in the United States between 2000 and 2010. Circulation 2013;128:2104–12. 24061087 10.1161/CIRCULATIONAHA.113.003862

[18] Baldi E , Conte G, Zeppenfeld K, Lenarczyk R, Guerra JM, Farkowski MM et al Contemporary management of ventricular electrical storm in Europe: results of a European Heart Rhythm Association Survey. Europace 2023;25:1277–83. 36196613 10.1093/europace/euac151 PMC10105853

[19] Chatzidou S , Kontogiannis C, Tsilimigras DI, Georgiopoulos G, Kosmopoulos M, Papadopoulou E et al Propranolol versus metoprolol for treatment of electrical storm in patients with implantable cardioverter-defibrillator. J Am Coll Cardiol 2018;71:1897–906. 29699616 10.1016/j.jacc.2018.02.056

[20] Willems S , Tilz RR, Steven D, Kääb S, Wegscheider K, Gellér L et al Preventive or deferred ablation of ventricular tachycardia in patients with ischemic cardiomyopathy and implantable defibrillator (BERLIN VT). Circulation 2020;141:1057–67. 32000514 10.1161/CIRCULATIONAHA.119.043400

[21] Al-Khatib SM , Stevenson WG, Ackerman MJ, Bryant WJ, Callans DJ, Curtis AB et al 2017 AHA/ACC/HRS guideline for management of patients with ventricular arrhythmias and the prevention of sudden cardiac death: a report of the American College of Cardiology/American Heart Association Task Force on Clinical Practice Guidelines and the Heart Rhythm Society. Circulation 2018;138:e272–391. 29084731 10.1161/CIR.0000000000000549

[22] Nguyen DT , Tzou WS, Sandhu A, Gianni C, Anter E, Tung R et al Prospective multicenter experience with cooled radiofrequency ablation using high impedance irrigant to target deep myocardial substrate refractory to standard ablation. JACC Clin Electrophysiol 2018;4:1176–85. 30236391 10.1016/j.jacep.2018.06.021

[23] Ene E , Nentwich K, Deaconu A, Berkovitz A, Halbfass PH, Sonne K et al Midterm results of bipolar ablation in patients with intramural substrate and recurrent VTs based on a single center experience. Europace 2023;25:euad122.319.

[24] Futyma P , Ciąpała K, Sander J, Głuszczyk R, Futyma M, Kułakowski P. Bipolar radiofrequency ablation of ventricular arrhythmias originating in the vicinity of his bundle. Circ Arrhythm Electrophysiol 2020;13:e008165. 32063033 10.1161/CIRCEP.119.008165

[25] Patel A , Nsahlai M, Flautt T, Da-Warikobo A, Lador A, Tapias C et al Advanced techniques for ethanol ablation of left ventricular summit region arrhythmias. Circ Arrhythm Electrophysiol 2022;15:e011017. 35917467 10.1161/CIRCEP.122.011017 PMC9388546

[26] Gunturiz-Beltrán C , Domínguez Mafé E, Pérez-Roselló V, Ribes F, Navarro-Manchón J, Bellver-Navarro A. Ethanol ablation via a coronary sinus branch as an effective option in recurrent ventricular tachycardia and epicardial inaccessibility. Europace 2023;25:1516–1516. 36730083 10.1093/europace/euad001 PMC10105839

[27] Cuculich PS , Schill MR, Kashani R, Mutic S, Lang A, Cooper D et al Noninvasive cardiac radiation for ablation of ventricular tachycardia. N Engl J Med 2017;377:2325–36. 29236642 10.1056/NEJMoa1613773 PMC5764179

[28] Hanson M , Futyma P, Bode W, Liang JJ, Tapia C, Adams C et al Catheter ablation of intramural outflow tract premature ventricular complexes: a multicentre study. Europace 2023;25:euad100. 10.1093/europace/euad100 PMC10228610 37096979

[29] Mueller J , Chakarov I, Halbfass P, Nentwich K, Ene E, Berkovitz A et al Adverse prognosis of patients with septal substrate after VT ablation due to electrical storm. JACC Clin Electrophysiol 2023;9:790–804. 36951814 10.1016/j.jacep.2023.01.012

[30] Liang JJ , Bogun F. Bipolar ablation for intramural ventricular tachycardia substrate: ready for prime time? Heart Rhythm 2020;17:1508–9. 32371180 10.1016/j.hrthm.2020.04.034

[31] Roca-Luque I , van Breukelen A, Alarcon F, Garre P, Tolosana JM, Borras R et al Ventricular scar channel entrances identified by new wideband cardiac magnetic resonance sequence to guide ventricular tachycardia ablation in patients with cardiac defibrillators. Europace 2020;22:598–606. 32101605 10.1093/europace/euaa021

[32] Jáuregui B , Soto-Iglesias D, Zucchelli G, Penela D, Ordóñez A, Terés C et al Arrhythmogenic substrate detection in chronic ischaemic patients undergoing ventricular tachycardia ablation using multidetector cardiac computed tomography: compared evaluation with cardiac magnetic resonance. Europace 2021;23:82–90. 33038230 10.1093/europace/euaa237

[33] Santangeli P , Muser D, Zado ES, Magnani S, Khetpal S, Hutchinson MD et al Acute hemodynamic decompensation during catheter ablation of scar-related ventricular tachycardia. Circ Arrhythm Electrophysiol 2015;8:68–75. 25491601 10.1161/CIRCEP.114.002155

[34] Vergara P , Tzou WS, Tung R, Brombin C, Nonis A, Vaseghi M et al Predictive score for identifying survival and recurrence risk profiles in patients undergoing ventricular tachycardia ablation. Circ Arrhythm Electrophysiol 2018;11:e006730. 30562104 10.1161/CIRCEP.118.006730 PMC6301075

[35] Martins A , Silverio Antonio P, Couto Pereira S, Brito J, Valente Silva B, Da Silva P A et al Is it possible to predict mortality and recurrence of VT after ablation? PAINESD risk score applicability vs new predictors. Europace 2022;24:1112–1118.35030257

[36] Lakkireddy D , Shenthar J, Garg J, Padmanabhan D, Gopinathannair R, Di Biase L et al Safety/efficacy of DOAC versus aspirin for reduction of risk of cerebrovascular events following VT ablation. JACC Clin Electrophysiol 2021;7:1493–501. 34393085 10.1016/j.jacep.2021.07.010

[37] Reek S , Burri H, Roberts PR, Perings C, Epstein AE, Klein HU et al The wearable cardioverter-defibrillator: current technology and evolving indications. Europace 2017;19:335–45. 27702851 10.1093/europace/euw180

[38] Glöcklhofer CR , Steinfurt J, Franke G, Hoppmann A, Glantschnig T, Perez-Feliz S et al A novel LMNA nonsense mutation causes two distinct phenotypes of cardiomyopathy with high risk of sudden cardiac death in a large five-generation family. Europace 2018;20:2003–13. 29947763 10.1093/europace/euy127

[39] van Rijsingen IAW , Arbustini E, Elliott PM, Mogensen J, Hermans-van Ast JF, van der Kooi AJ et al Risk factors for malignant ventricular arrhythmias in lamin A/C mutation carriers. J Am Coll Cardiol 2012;59:493–500. 22281253 10.1016/j.jacc.2011.08.078

[40] Rootwelt-Norberg C , Christensen AH, Skjølsvik ET, Chivulescu M, Vissing CR, Bundgaard H et al Timing of cardioverter-defibrillator implantation in patients with cardiac laminopathies—external validation of the LMNA-risk ventricular tachyarrhythmia calculator. Heart Rhythm 2023;20:423–9. 36494026 10.1016/j.hrthm.2022.11.024

