# Supplementary material for: Management of ventricular tachycardias: insights on centre settings, procedural workflow, endpoints, and implementation of guidelines—results from an EHRA survey
Source: Europace. 2024 Feb 14;26(2):euae030. doi: 10.1093/europace/euae030 (PMC10872712; doi:10.1093/europace/euae030)
Supplement: euae030_Supplementary_Data [file euae030_supplementary_data.pdf]

EHRA European Heart Rhythm Association

# Survey on VT Ablation_2

## Introduction

**Dear colleagues,**
**The European Heart Rhythm Association (EHRA) is exploring the way ventricular ablation is managed and the types of procedural methods which are used. To help us in this endeavour, please complete a short survey consisting of 2 parts:**

**Part A emphasises on general aspects of ventricular ablation at your centre.**

**In Part B understanding of your specific procedural settings in VT ablation.**

**On behalf of the EHRA Scientific Initiatives Committee (SIC) we thank you for participating your time and effort.**

* 1. GDPR Disclaimer

We will not disclose your identity to any third party.

We comply with the European General Data Protection Regulation (GDPR) 2016/679. Any personal data processed in connection with this survey will be treated confidentially and only used by the ESC for the purposes of market research and not for promotion. Survey results will be kept for a maximum of 48 months for analysis and quality control purposes. We take all reasonable care to prevent any unauthorised access to your personal data. We respect your privacy and your right to access, modify, or remove your personal data. At any time, you can ask to know what personal data is being held. If you have any questions about data protection or require further information, please contact our data protection officer (DPO) at dpo@escardio.org.

You have the right to end your participation in this survey at any time.

Please confirm that you have read the above and agree to participate in this survey.

- ◯ yes
- ◯ no

EHRA European Heart Rhythm Association

Survey on VT Ablation_2

## General aspects (Part A)

**Baseline data for your center**

2. What is your country?

[ ▾ ]

[ ]

3. How many ablations overall are performed at your centre/year?

- <50
- 50-100
- 100 – 300
- 300 – 900
- >1000 / y

4. In which hospital type do you work?

- University hospital
- Non-University Hospital
- Private center
- Other (please specify)

5. Number of physicians performing VT ablation on a regular basis?

6. How many VT ablation/year (including outflow tract VT) are performed?

7. Which percentage of your VT ablation is performed in patients with ICM?

0 100

8. Is heart surgery available onsite?

- Yes
- No

9. Are support systems (ECMO, Impella etc.) available?

- Yes
- No

10. Do you have a dedicated VT storm unit / 24h VT service/expertise?

- Yes
- No



## Typical procedural settings for VT ablation at your centre

11. Do you routinely perform  pre-procedural imaging?

- [ ] No
- [ ] Yes, CMR/LGE CMR
- [ ] Yes, CT/3D CT including coronary angiogram
- [ ] Please specify software

12. Intraprocedural visualisation aspect - Do you integrate obtained imaging into intraprocedural electroanatomical mapping?

- ( ) Yes
- ( ) No
- ( ) None of the above

13. If ICE is available when do you use it?

14. Do you perform coronary angiography before VT ablation?

- ( ) Yes
- ( ) No

15. Procedure is generally performed in:

| | Yes | No |
|---|---|---|
| Deep analgo-sedation, if tolerated | ( ) | ( ) |
| Always general anesthesia | ( ) | ( ) |

16. What is your favored medication/treatment for acute treatment in VT or VT storm (multiple answers possible, please indicate ranking if answered multiple)

- Amiodarone (de novo or escalation)
- Lidocaine
- Ajmaline
- Mexiletine
- Non-selective ß-Blocker
- stellate ganglion blockade/sympathectomy
- Intubation and deep sedation

EHRA European Heart Rhythm Association

Survey on VT Ablation_2

## Pre and Post Ablation

17. Do you stop (D)OAC/DAPT before procedure

- Yes
- No

18. If you stop, how long before procedure (in hours)?

0 48

19. What is your (D)OAC/Antiplatelet of choice after ablation (no AF indication)?

20. How long do you prescribe (D)OAC/Antiplatelet (in weeks)?

0 52

EHRA European Heart Rhythm Association

Survey on VT Ablation_2

## What is your AAD regime and/or use of LifeVest post ablation

21. Do you always stop AAD after successful ablation

- ◯ Yes
- ◯ No
- ◯ Sometimes

22. Do you continue AAD for at least 3 month after successful ablation?

- ◯ Yes
- ◯ No
- ◯ Sometimes

23. Do you use LifeVest in patients with preserved LV-Fx after VT ablation?

- ◯ Yes
- ◯ No

24. When do you perform VT ablation in “first do” procedures in ischemic cardiomyopathy patients (ICM)?

- ☐ After the first shock
- ☐ After multiple shocks
- ☐ Increasing nsVT or ATP episodes
- ☐ “Prophylactic ablation” irrespective of ICD intervention

25. When do you perform VT ablation in “first do” procedures in Non-ICM?

- ☐ After the first shock
- ☐ After multiple shocks
- ☐ Increasing nsVT or ATP episodes
- ☐ “Prophylactic ablation” irrespective of ICD intervention

26. Based on what do you decide to use an assist device for VT ablation?

- ☐ I-VT Score/PAINESD Score
- ☐ Patient in VT storm
- ☐ Severely impaired LV function

27. In case of ablation failure does your centre have access to any of the following advanced ablation technique listed?

- [ ] Epicardial ablation
- [ ] Alcohol ablation
- [ ] Bipolar ablation
- [ ] Half-normal-saline/dextrose-5 in water (D5W) irrigation
- [ ] Stereotactic radiotherapy
- [ ] arrhythmia surgery
- [ ] Not available onsite but cooperation and/or referral option in my country
- [ ] Not available in my country
- [ ] All that applies

28. What do you use for vascular access and access closure?

- [ ] US-guided puncture
- [ ] Z-suture for venous access
- [ ] Closure device (e.g. AngioSeal) for arterial access
- [ ] Closure device for venous access
- [ ] Manual compression only

29. In which cases do you perform genetic testing?

- [ ] In all DCM/HNDCM cases
- [ ] In DCM/HNDCM patients with family history of DCM/HNDCM or SCD (<50 years)
- [ ] In DCM/HNDCM patients with AV conduction abnormalities
- [ ] I do not consider genetic testing valuable in this setting

30. Do you perform EP Study for VT/SCD risk assessment?

- [ ] No
- [ ] Only in patients with first SMVT episode
- [ ] Only in symptomatic (syncope/dizziness) patients with known SHD

EHRA European Heart Rhythm Association

Survey on VT Ablation_2

## Procedural specifics (Part B)

31. How do you obtain access to the LV in the vast majority of your cases?

- ( ) Always retrograde aortic and transseptal access
- ( ) Starting retrograde aortic access only
- ( ) Starting transseptal access only

32. When do you opt for an epicardial access beforehand (procedure start) ?

- [ ] Only in Redo cases
- [ ] Only in NICM
- [ ] In NICM during first procedure
- [ ] Only based on ECG criteria

33. How would you describe your procedural workflow (multiple answers possible)?

- [ ] Electro-anatomical mapping and VT induction attempt
- [ ] High-Density Mapping routinely
- [ ] No induction attempt, substrate-based ablation (HD map/MRI/CT based)
- [ ] ILAM-, DEEP-, LAVA- mapping guided ablation
- [ ] Other (please specify)

34. Which mapping catheter do you use for electro-anatomical mapping?

- [ ] High density mapping catheter
- [ ] Mapping via ablation catheter
- [ ] None, only CT or MRI
- [ ] Other (please specify)

35. From which cavity do you perform your EPS for VT induction?

- ○ RV and/or RVOT only
- ○ LV only
- ○ RV and LV

36. Your procedural endpoint and lesion assessment consists of (multiple answers possible)

- [ ] Endpoint of non-inducibility for VT
- [ ] Abolishment of all LAVAs
- [ ] Rendering substrate non-excitable (pacing with 10mV/2ms)
- [ ] Impedance drop >20 Ohm
- [ ] Abolishment of all long Stim-QRS pacing sites (>70 ms)/de-channeling
- [ ] Ablation of clinical VT only
- [ ] Core isolation/encirclement
- [ ] Reduction of local EGM voltage >90%
- [ ] Combination of all

37. What type of catheter do you use and what is your typical (standard) power setting?

- [ ] Irrigated contact force RF cathete
- [ ] Irrigated non-contact force RF
- [ ] 30 - 50W
- [ ] >50 W
- [ ] >60 W
- [ ] PFA
- [ ] Ultra-Cryo
- [ ] Max. power used epicardially

38. Do you use ablation index or lesion size index (knowing its validation for LA only)?

- ( ) Yes
- ( ) No



39. What is you target AI value (your max. value please)

40. What it your target LSI value (your max. value please)

41. What is your AI value ?

0 999

42. What is your LSI value ?

0 999

EHRA
European Heart
Rhythm Association

Survey on VT Ablation_2

43. When do you perform advanced ablation techniques (e.g. bipolar - please see Part)?

- [ ] Only in case of repeat ablation after initial failure (second procedure)
- [ ] In first procedure if VT documentation or disease entity implies (e.g. NICM)
- [ ] Not available in my centre

44. If needed, which assist device do you use the most frequently?

- ○ Impella
- ○ ECMO
- ○ IABP
- ○ Combination (ECMO/Impella)

EHRA European Heart Rhythm Association

## Survey on VT Ablation_2

### Thank you

**Dear colleague,**

**Thank you very much for completing the survey.**

**Your input is very much appreciated and will provide us with invaluable insights.**

**Please click on “Done” to submit your responses.**
